# Danaparoid—Consensus Recommendations on Its Clinical Use

**DOI:** 10.3390/ph17121584

**Published:** 2024-11-25

**Authors:** Rupert M. Bauersachs, Edelgard Lindhoff-Last, Robert Klamroth, Andreas Koster, Marc Schindewolf, Harry Magnani

**Affiliations:** 1 Cardioangiology Center Bethanien, Vascular Center and Coagulation Center, Im Prüfling 23, 60389 Frankfurt, Germany; e.lindhoff-last@ccb.de; 2 Center for Vascular Research, Munich, Hochkalter Strasse 4a, 81547 Munich, Germany; 3 Department Angiology and Haemostaseology, Vivantes Klinikum Friedrichsheim, Landsberger Allee 49, Friedrichshain, 10249 Berlin, Germany; robert.klamroth@vivantes.de; 4 Department of Cardio-Anaesthesiology, Sana-Herzzentrum Cottbus GmbH, Leipziger Straße 50, 03048 Cottbus, Germany; andreas.koster@sana.de; 5 Division of Angiology, Swiss Cardiovascular Center, Inselspital, Bern University Hospital, University of Bern, CH-3010 Bern, Switzerland; marc.schindewolf@insel.ch; 6 Independent Researcher, 5345 MT Oss, The Netherlands; harry.magnani@hetnet.nl

**Keywords:** Orgaran, danaparoid, heparinoid, anticoagulant, dosing, monitoring, heparin-induced thrombocytopenia, off-label use, thromboprophylaxis, venous thromboembolism

## Abstract

(1) Background: Danaparoid sodium is a heparinoid antithrombotic that has been used for over 40 years for prophylaxis of DVT in non-HIT patients and for the treatment of heparin-induced thrombocytopenia (HIT) with and without thrombosis. This update summarises current information on its pharmacology and reviews danaparoid dose management in a broad spectrum of clinical situations, including off-label indications. (2) Methods: Evidence from published clinical studies, case reports, compassionate use of danaparoid, and spontaneously reported serious adverse events is summarised and analysed by an interdisciplinary expert group to develop a consensus on dosing regimens of danaparoid for complex clinical situations, including vulnerable patient populations. (3) Results: Dosing regimens are proposed, together with monitoring recommendations and target anti-factor Xa ranges. (4) Conclusion: In a comprehensive summary detailed interdisciplinary dosing recommendations are described to provide a basis for safe and effective use of danaparoid.

## 1. Introduction

### 1.1. Danaparoid Sodium

Danaparoid sodium, marketed as Orgaran^®^, is a heparinoid antithrombotic drug, that was developed as an alternative to heparin in 1977. Since that time, it has been used in many clinical indications. In 1991 danaparoid was approved in many countries for venous thromboembolism (VTE) prophylaxis in adults undergoing orthopaedic, major abdominal or thoracic surgery, and for thromboprophylaxis in non-haemorrhagic stroke. In 1994, it was licensed for the treatment of acute heparin-induced thrombocytopenia (HIT) and past or remote HIT (when HIT assays have become negative), and other clinical situations with suspected heparin-intolerance, e.g., delayed-type hypersensitivity (DTH) to heparin and heparin resistance. After approval for DVT prophylaxis in adults in the US in 1997, it was licensed in 2000 in Japan for the treatment of disseminated intravascular coagulation (DIC) in adults and in children. Within the spectrum of HIT patients, danaparoid was also allowed for women in pregnancy, in children, and in renal failure patients, provided that no other suitable antithrombotic was available. As a result, danaparoid has also been extensively used off-label as an anticoagulant for not only similar non-HIT patients with some form of heparin-intolerance, but for patients requiring anticoagulation as part of the management of hepatic, inflammatory, and immune-disorders.

While dosing information is available in the current European Summary of Product Characteristics (SmPC) for approved indications, there are considerable variations, both within and between countries, concerning the optimal dosing intensity and administration route as judged by published case reports. This is partly due to the various clinical scenarios patients are presenting with, particularly if they have HIT or are treated in an intensive care unit (ICU). Off-label use of danaparoid is frequent and, because dosing recommendations are sparse, dosing is often adapted from an approved indication, which may not be optimal for the intended off-label clinical situation. The present article therefore focuses on a consensus of dosing recommendations for both its approved and off-label uses and aims to provide guidance for critical clinical situations. These recommendations are based on more than 40 years of published and personal experience. Nevertheless, the treating physician always needs to balance benefit and risk for the individual patient and situation.

### 1.2. Heparin-Induced Thrombocytopenia

Heparin-induced thrombocytopenia is a life- and limb-threatening immune-mediated, prothrombotic disorder classically occurring after heparin exposure in about 2.6% and 0.2% after UFH and LMWH, respectively [1,2]. Antibodies against platelet-factor 4 bound to heparin can cause this severe clinical syndrome with thrombocytopenia, arterial, and/or venous thrombosis. Rapid diagnosis, immediate discontinuation of heparin, and initiation of non-heparin anticoagulation are crucial in the management of HIT [2]. Current reviews [2,3,4,5] and guidelines [6,7,8] summarise epidemiology, the complex pathophysiology, diagnostic workup, and therapeutic management options of HIT.

## 2. Chemistry and Pharmacology

Danaparoid (sodium) is a natural mixture of short chain linear glycosaminoglycans (GAGs) that does not include heparin. It consists predominantly of heparan sulphates (HS, 84%) [9,10], of which about 5%, the HA–HS, has High Affinity (HA) for antithrombin (AT) but the major component, the NA–HS, has No Affinity (NA) for AT. The remainder is dermatan sulfate (DS, about 12%) and a small percentage of chondroitin sulphates [11]. The molecular weight average of the HS, which provides the main activities of danaparoid (see below) is 3.3 to 3.5 kDa [12].

The HA–HS chains share the identical AT-binding pentasaccharide sequence with heparins and fondaparinux. Enzyme hydrolysis of danaparoid provides disaccharides the large majority of which consist of glucuronic acid (in place of heparin’s iduronic acid 2-sulphate) and N-acetyl glucosamine (in place of heparin’s glucosamine N-sulphate). Hence, danaparoid has a lower overall degree of sulphation and negative charge density than heparins.

In vitro pre-treatment cross-reactivity studies with danaparoid have shown means of 5.2% to 7.6% of fresh and archived HIT sera [13,14]. The range of 0% to 50% depends upon the sensitivity of the method, being lowest for the platelet aggregation test (PAT), higher for routine platelet activation assays (HIPA and SRA) and highest for an experimental fluid-phase PF-4 immunoassay [15]. A possible explanation for these results is that the chains of the 5% by weight HA–HS sub-fraction, unlike the remainder of danaparoid, include the AT-binding pentasaccharide sequence. This gives the HA–HS a charge density similar to that of the heparins and, when isolated, this sub-fraction was found to cross-react 100% with HIT antibodies; however, on adding the remainder of danaparoid (i.e., mainly consisting of the NA–HS) this cross-reactivity was completely blocked [16]. The most likely cause of pre-treatment cross-reactivity, however, appears to be due to residual heparin in the sample [17,18,19]. Two studies examined the clinical relevance of a positive pre-treatment danaparoid cross-reactivity test [15,20] and found that in almost all cases there were no clinical consequences. Although the clinical relevance of these in vitro results remained unknown, testing for in vitro cross-reactivity before the start of danaparoid treatment is no longer considered necessary [21].

**Figure 1:**
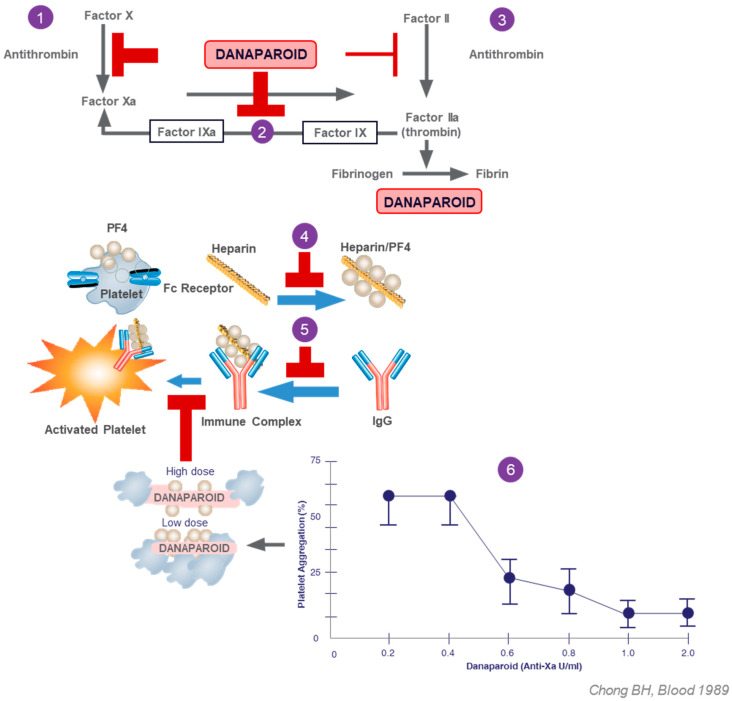
Danaparoid sodium in the treatment of acute HIT: multiple modes of action [24].

Danaparoid at very low concentrations can increase PF4 binding to platelets. At therapeutic concentrations, danaparoid inhibits both the formation of PF4–heparin complexes, binding of PF4 and of PF4–heparin complexes to cell surfaces, and removes PF4–heparin complexes from cell surfaces, essentially by disrupting these complexes. Danaparoid has also been shown to inhibit HIT-induced platelet activation and thromboxane B2 release in a dose dependent manner (see Figure 1) [10,17,22,23]. 

Figure 1 summarizes two principal modes of action of danaparoid in the treatment of acute HIT including the action on the coagulation cascade (1 to 3) and immunomodulation actions (4 to 6), respectively. Within the coagulation cascade danaparoid acts by indirect (via AT activation) inhibition of factor X (1) activation, and by direct inhibition of thrombin activation of Factor IX (2) thus inhibits thrombin-induced platelet aggregation (2), leading to effective inhibition of thrombin generation and thrombus formation. As a result this also limits platelet aggregation (3). Immunomodulatory actions include the interference with PF4–heparin complex formation (4) and the binding of HIT antibodies to PF4/heparin complexes (5). These actions also require therapeutic danaparoid dosing administered as a continuous iv infusion [1]. The inhibition by danaparoid of immune-mediated platelet activation has been shown to be dose dependent (see insert (6), supporting the rationale for therapeutic dosing intensity even if no thrombosis is present.

By these mechanisms it prevents platelet activation by HIT antibodies and, thus, inhibits HIT pathogenesis [23,24,25]. All these actions can be attributed to the presence of the NA–HS sub-fraction, which at therapeutic doses totally overcomes the higher overall negative charge of the HA–HS that allows the latter to bind to PF4 [22].

Hence, in vivo cross-reactivity (i.e., sero-conversion with a positive test) has rarely been described [10,23,25,26,27] and, in some patients with early complications, the possibility of pre-existing heparin in either the patient or the sample tested for cross-reactivity or inadvertent heparin administration had not been considered. Some patients with pre-treatment positive tests have successfully received danaparoid. About half the former patients, and only a few of the latter, developed what were considered to be clinical sequelae; e.g., after 3 to 5 days of danaparoid therapy, the platelet count failed to increase and/or a new thrombotic event occurred. These events were also noted in about 13% of patients who were not tested before or after exposure to danaparoid. An indirect comparison [28] of the clinical outcomes of HIT patients in major studies treated with danaparoid or two thrombin inhibitors (argatroban and hirudin) showed that, compared with their respective controls, danaparoid performed better for both the composite efficacy events (new thromboses or extension, all-cause mortality, and amputation) and for major bleeding. Since neither of the two thrombin inhibitors could possibly cross-react with heparin-induced HIT antibodies, it is unlikely that these events are associated with danaparoid clinical cross-reactivity. It is more likely that failure to respond to danaparoid treatment is related to one or more of the following: the natural progression of HIT, inadequate treatment intensity [29], delayed initiation of danaparoid therapy, inadvertent heparin co-administration, sepsis or other severe co-morbidity, delayed onset HIT [30], or co-medication.

### 2.1. Pharmacodynamics

Danaparoid inhibits thrombin generation indirectly via AT to inhibit factor Xa [11], and directly by inhibiting thrombin activation of Factor IXa [31]. It also shows minor indirect antithrombin activity via both AT and Heparin cofactor II [11] (see Figure 1). The combined result is efficient inhibition of thrombus formation and extension [11]. Danaparoid hardly affects platelet activity [32], hence primary haemostasis remains intact [11]. Although it does not appear to influence the fibrinolytic system [33], it makes fibrin clots more permeable to thrombolytics [34] in vitro, which may explain the delayed post-operative bleeding after the high doses required for cardio-pulmonary bypass surgery [35].

Danaparoid does not cross guinea pig [36] or human placentae [37], and is not secreted into breast milk in clinically relevant amounts [38]. It shows a flat dose–response curve with clotting tests [39] (activated partial thromboplastin time (APTT), prothrombin time (PT), thrombin time (TT), and activated clotting time (ACT) at recommended therapeutic dosing levels; hence, these test are unsuitable for monitoring purposes. The overall low degree of sulphation and negative charge of danaparoid explain its lack of binding to heparin-binding proteins, except to its clotting cascade targets, thus providing almost 100% bioavailability compared to 30% to 80% of heparins. Importantly, this high bioavailability means that 1 U of danaparoid is not equivalent to 1 U of the heparins. Thus, the calibration curve for monitoring plasma levels of danaparoid induced anti-Xa activity (by chromogenic or Heptest methods) must be constructed with danaparoid (not any of the heparins or their international standards). In such an assay, danaparoid shows a linear dose-response. Failure to use danaparoid calibration samples can lead to overestimation of its plasma level and, if subsequent attempts are made to reduce this spurious high level by reducing danaparoid doses, thrombotic complications may occur.

In different animal models and a few small human studies, therapeutic doses of danaparoid have shown immune-modulating and anti-inflammatory effects [40,41,42] that appear to be both dependent and independent of its antithrombotic activity [40]. In addition, it has a unique ability to inhibit the pathogenesis of HIT [23] (see Figure 1) and platelet aggregation by HIT antibodies [24]. These effects support its use for the treatment of HIT, DIC [43], hepatic microangiopathies [44], and infections such as COVID-19 [45]; they are particularly attributed to the NA–HS component of danaparoid.

### 2.2. Pharmacokinetics

With both i.v. and s.c. bolus injection, the amidolytic plasma anti-Xa activity shows a linear dose response due to the high bioavailability of danaparoid. Because of its multi-component nature, there is no single half-life of danaparoid. The half-lives of elimination of anti-Xa and thrombin generation inhibiting (TGI) activities are approximately 25 h and 7 h, respectively [46]. However, in animal models, only the half-life of its TGI effect, integrating the activities of all danaparoid constituents on haemostasis, approximates to the clinically important antithrombotic half-life of danaparoid [11]. The assumption that the same relationship holds for humans is supported by early clinical dose-finding studies that showed twice-daily danaparoid dosing was significantly more effective than once-daily dosing for DVT prophylaxis [47,48]. Steady-state levels of plasma anti-Xa activity are usually reached within 4–5 days after both s.c. and i.v. intermittent injections but, when measured by TGI activity, steady-state levels are already reached within 1–2 days.

Administration route, age, sex, and race do not seem to affect danaparoid PK parameters [46,49,50]. Danaparoid is mainly eliminated renally [11,40,50] and the half-life of plasma anti-factor Xa activity has been found to be prolonged by up to 50% in severely impaired renal function, and in renal failure requiring dialysis [51,52]. The liver is not primarily involved in its elimination or metabolism [49], but the plasma anti-Xa activity drops considerably between intermittent haemodialysis [52,53] suggesting possible action of circulating endogenous heparanases.

### 2.3. Antidote

Danaparoid has no direct antidote, and heparin antidotes, like protamine sulphate, are not effective. Although its anticoagulant effects may be reversed with recombinant factor FVIIa (90 µg/kg iv), as has been suggested [34,54], clinical use of recombinant factor VIIa has not been reported so far. Plasmapheresis can remove danaparoid from the circulation [55], and fresh frozen plasma (FFP) transfusions may dilute its effect.

### 2.4. Pharmacy

Danaparoid is supplied as Orgaran^®^ in clear glass, break-neck ampoules containing 750 U danaparoid sodium in 0.6 mL sodium chloride for subcutaneous or intravenous administration. The ampoule also contains sodium bisulphite as a preservative. The ampoules should be stored in the dark, preferably refrigerated. The trade name Orgaran^®^ is used throughout Europe and English-speaking countries. In Japan it is referred to as danaparoid sodium in all publications. In the US, Organon, Inc. (West Orange, NJ, USA) withdrew Orgaran^®^ on 14 August 2002 due to a shortage in drug substance by the manufacturer [10,56].

#### Preparation of Therapeutic Infusion

The Orgaran^®^ stock solution for i.v. infusion is made by adding three ampoules (2.250 U) Orgaran to 250 mL infusion fluid (either saline (0.9%), dextrose (5%), dextrose–saline, Ringer’s, lactated Ringer’s, and 5% mannitol) to provide 9 U/mL. Table 1 provides the stock solution infusion rates required to deliver the desired amount of danaparoid per hour for the step-down infusions of the therapeutic regimen. The stock solution can be kept for up to 48 h if refrigerated at 4–10 °C.

## 3. Approved Indications

### 3.1. General Dosing Regimens

Pharmacokinetic modelling, clinical development studies, and experience with the compassionate use program (CUP) were the basis for the danaparoid dosing regimens in both non-HIT and HIT patients. Note that, unless otherwise indicated, acute and subacute HIT patients are considered together as ‘*current HIT*’ patients with respect to danaparoid dosing, as both situations require treatment initiation at therapeutic dosing independent of a thrombotic event [57,58]. This is because many HIT patients have clinically asymptomatic thrombosis at presentation, which may become clinically evident a few days later [59], and the risk of thrombosis development is elevated until the level of the specific HIT antibodies (Ab) become undetectable by routine immunoassays (usually by 120 days after acute HIT onset).

Furthermore, comparative studies have revealed that unless current HIT patients require an immediate operation or invasive vascular procedure, their therapeutic danaparoid treatment should be given as a continuous i.v. infusion for at least one to two weeks. Danaparoid is one of the antithrombotics recommended for management of all phases of HIT [7,8], especially because it appears to reduce the need for limb amputations compared with argatroban [8], the only other generally approved antithrombotic for the management of HIT. Using the above sources of information, the target plasma anti-Xa activity ranges for an optimal efficacy/safety ratio were found to be 0.1–0.4 U/mL for thrombosis prophylaxis and 0.4–0.8 U/mL for thrombosis treatment.

#### 3.1.1. The Danaparoid Loading Dose

When therapeutic danaparoid treatment is started a loading bolus is essential for immediate achievement of the desired plasma anti-Xa activity target range as soon as possible. Therefore, unless there are contradicting reasons, the bolus injection should not be omitted [5], but could be reduced if felt necessary, e.g., if the bleeding risk is high and/or the patient recently had a spinal or epidural catheter removed (see below).

#### 3.1.2. Continuous Infusion of Danaparoid

The continuous infusion of danaparoid (Regimen 2, see Table 2) ensures that all components of danaparoid are present in the circulation at all times. The two step-down infusions prior to the maintenance infusion are crucial, because otherwise an early dip in plasma anti-Xa activity might occur [60] (blue line in Supplementary Figure S1), during which antithrombotic protection (and, in HIT patients, the ability of danaparoid to neutralise the effect of the HIT-Ab) are diminished within the first 24 h. The need to maintain constant levels of its active components at all times is related to their widely differing elimination half-lives. With intermittent s.c. or i.v. injections, there is loss of the immune-modulating/anti-inflammatory and some antithrombotic activity before the next injection; whereas the infusion regimen provides constant presence of these different activities, thus optimising the danaparoid efficacy. The ideal plasma anti-Xa activity response during the initiation and subsequent maintenance danaparoid infusions is shown by the red line in Supplementary Figure S1.

#### 3.1.3. Monitoring Using the Plasma Anti-Xa Activity

At the time of its development, the only sufficiently sensitive method available for monitoring danaparoid was the plasma amidolytic anti-Xa activity assay which has continued to be used to this day. Note, however, that the half-life of the plasma anti-Xa activity is only a surrogate for the half-life of danaparoid (see above).

The danaparoid dosing regimen required to achieve the therapeutic target range of 0.4–0.8 anti-Xa activity/mL plasma is mainly based on PK modelling for subjects with an acute ischemic stroke [60], supported by data from the compassionate use program [13] treatment of VTE [61] and studies in intensive care patients [29,62,63]. Steady-state anti-Xa activity levels are reached immediately after the recommended i.v. loading dose and infusion rates (for details see Table 2, regimen 2a); hence, blood samples for monitoring can be taken at any time. However, if therapeutic s.c. doses are administered, monitoring should be performed 3–4 h after an injection. Treatment failures documented in HIT patients were frequently due to inadequate dosing [29]. The same plasma anti-Xa activity range was also used for severely ill renal failure subjects undergoing continuous renal replacement therapy (CRRT) [64].

Routine monitoring is not required during administration of the therapeutic Regimen 2a. However, it is recommended for detecting danaparoid under-dosing, i.e., if plasma anti-Xa activity remains persistently less than 0.4 U/mL in subjects weighing > 90 kg, and especially the morbidly obese [65], or to detect danaparoid accumulation, i.e., >0.8 U/mL in children, underweight adults, or patients with severe renal failure (eGFR < 15 mL/min/1.73 m^2^).

In general, plasma anti-Xa activity levels during danaparoid treatment correlate poorly with thrombotic or bleeding events unless they are well outside the target ranges at steady-state [35]. Therefore, it is advised to use the recommended dose (Table 2) with clinical monitoring for bleeding or thrombosis, and to use anti-Xa monitoring in at-risk subjects to detect over- or under-dosing, respectively, or in case of bleeding or thrombotic problem. If the plasma anti-Xa activity on Day 3 is below the target range, the infusion rate should be increased by 50 U/h and anti-Xa activity measured again 24 h later. If necessary, repeat until the target range is achieved, but do not exceed 200 U/h. If on Day 3 the plasma anti-Xa activity is above the target, the maintenance infusion rate should be lowered by 50 U/h and anti-Xa activity measured again 24 h later. If necessary, repeat until the target range is achieved.

In clinically stable patients, there is no need for further laboratory monitoring once the target range is achieved. However, laboratory monitoring should be performed every other day in clinically unstable patients, particularly if at risk of deteriorating renal function to avoid bleeding caused by danaparoid accumulation.

The specific dosing regimen for different clinical scenarios are summarised in Table 2, which are for guidance only.

### 3.2. Special Dosing Considerations

#### 3.2.1. Danaparoid Dosing in the Elderly

Pharmacokinetic studies showed no differences compared to young subjects despite the age-related decrease in renal function. Phase II/III clinical studies for post-operative DVT prophylaxis or ischemic stroke included nearly 4000 non-HIT patients, and the CUP another 688 mainly HIT patients, of whom > 60% and >50%, respectively, were 65 years or older (range 14–101 years). The results overall show that there were no differences in efficacy or safety outcomes between patients at and above 65 years and compared to patients under 65 years, respectively. Thus, all the above dosing recommendations apply for patients up to 95 years, but adaptation of the dosages may be necessary if their eGRF is below 15 mL/min/1.73 m^2^.

#### 3.2.2. Renal Failure Requiring Extracorporeal Circulation

Danaparoid should be used with caution in patients with moderate to severe renal dysfunction and impaired haemostasis because the half-life of its anti-Xa activity response is prolonged. However, for extracorporeal support special dosing regimens have been established to maintain safe circuit patency while preventing Danaparoid accumulation.

Severely ill acute HIT subjects may develop acute renal failure requiring either intermittent haemodialysis (HD) or continuous renal replacement therapy (CRRT) and some non-HIT subjects may develop HIT during use of one of these extracorporeal circuits (ECC). Danaparoid has been shown to be a suitable anticoagulant to maintain patency of the ECC [53,66,67,68,69] provided that the dosing regimens shown in Table 2 (regimen 4a/4b) are applied. Note that heparin-bonded ECC sets or catheters should not be used for HIT subjects requiring CRRT or haemodialysis.

#### 3.2.3. Neuraxial (Spinal/Epidural) Anaesthesia and Lumbar Puncture

During routine peri-operative controlled randomised trials (CRTs) for DVT prophylaxis, danaparoid treated patients received at least one 750 U s.c. dose (the last 1–4 h) prior to surgery. The published studies with detailed information [70,71,72,73] included 854 danaparoid treated patients and, of these, 329 received neuraxial (spinal or epidural) anaesthesia alone or in combination with general anaesthesia. No related adverse events were recorded.

Nevertheless, danaparoid received a black-box warning in the US stating ”neuraxial anaesthesia or lumbar puncture in patients on or expecting to be given prophylactic use of heparins and heparinoids, may be associated with epidural or spinal haematoma resulting in prolonged or permanent paralysis. The risk could be increased by the prolonged use of a peridural or spinal catheter for analgesia, by the concomitant use of drugs affecting haemostasis such as nonsteroidal anti-inflammatory drugs (NSAIDs), and by traumatic or repeated puncture”.

According to current evidence-based recommendations for anticoagulation in the patients provided with neuraxial anaesthesia, danaparoid should only be given post-operatively and avoided with the use of spinal catheters [74,75].

#### 3.2.4. PCI (Percutaneous Coronary Intervention) and Angioplasty

In HIT patients danaparoid can be used for cardiac catheterisation and/or PCI (with or without stenting) as an adjunct to antiplatelet therapy. The pre-intervention i.v. bolus dose (2250 U or 3750 U, if the patient weighs more than 90 kg) is the same for both procedures, but if given for catheterisation and the angioplasty follows within 4 h then the i.v. bolus does not need to be repeated.

If necessary, an i.v. infusion of 150–200 U/hour for 1–2 days can be started immediately after the PCI. After several days of i.v. maintenance therapy the patient may either be switched to alternative oral anticoagulants, as indicated above, or to the subcutaneous DVT prophylaxis Regimen 1.

#### 3.2.5. Cardiopulmonary Procedures with Bypass

Cardiac surgery with cardiopulmonary bypass (CPB) requires ultra-high dose anticoagulation corresponding to heparin dosages of 400–500 U/kg. However, in the case of heparin use, its anticoagulant effect is reversed after the CPB procedure via protamine. Post-operative bleeding with the need for re-operation occurs in 2% of patients, even when only lower-risk procedures are included in the analysis [76]. However, in high-risk procedures, such as implantation of a left ventricular assist device, 14% of patients needed re-operation due to excessive bleeding [76,77]. Due to lack of other potent intravenous anticoagulants other than heparin, danaparoid has not been used for anticoagulation of HIT patients in cardiac surgery since the end of the 1990s. However, the pharmacology of danaparoid renders this agent not as first line agent in this high-risk setting. The elimination half-life is longer than that of heparin and even prolonged in cases of renal impairment. No antidote is available and options for point of care monitoring of the anti-Xa activity in the operation room are limited. In the absence of an antidote, the use of danaparoid in cardiac surgery was associated with a high risk of bleeding complications [35,78]. With the introduction of the short-acting direct thrombin inhibitor bivalirudin in cardiac surgery at the beginning of 2000, the use of danaparoid in cardiovascular surgery decreased. Current HIT guidelines clearly favour the use of bivalirudin or other alternative anticoagulation strategies/procedures in this high-risk setting [8].

#### 3.2.6. Danaparoid Anticoagulation During Pregnancy and Postpartum

Animal studies did not demonstrate teratogenic effects, adverse effects on fertility, or placental transfer of danaparoid [36]. The use of danaparoid in pregnancy and/or post-partum has not only been indicated in the context of HIT, but also in patients with DIC, thrombophilia, recurrent pregnancy loss in the context of antiphospholipid syndrome, and especially with heparin intolerance, mostly due to allergic cutaneous reactions, i.e., delayed-type hypersensitivity reactions (DTH). Hence, its use has been reported in 206 pregnancies [79,80,81,82,83,84].

Currently, there are only limited data from single and multiple case reports, and small randomised controlled trials are available of danaparoid use in pregnancy. Nevertheless, it is recommended for use in pregnancy if a substitute for unfractionated heparin is required [7,85,86]. Details of the danaparoid treatment during and after pregnancy are shown in Table 3. Most of the subjects with a current, past, or family history of TE, irrespective of their HIT status, received therapeutic dosages, either by danaparoid infusion or by high subcutaneous doses to allow for self-injection at home. In the six cases in which umbilical cord blood was tested for placental transfer of danaparoid, no anti-Xa activity was found [79]. In some pregnancies, the danaparoid dosing regimen was adapted during long term use as clinical circumstances changed, e.g., initial therapeutic danaparoid for acute thrombosis was switched to lower prophylactic levels later on, or an increase from prophylactic to therapeutic dosing was used to manage a new thrombotic event. In a review of 91 danaparoid treated pregnancies [37], 30 patients had an acute HIT, 17 were post HIT, and 44 had a non-HIT indication for danaparoid treatment. In each of the groups, venous thromboembolic events occurred in five (16.7%), zero (0%), and zero (0%) patients, respectively. Bleeding events occurred in two (6.7%), two (11.8%), and one (2.3%) patient, respectively. The low maternal and foetal adverse event frequencies of thrombotic and bleeding events and other pregnancy complications indicate that danaparoid can be an effective and safe alternative antithrombotic in pregnancies complicated by HIT or heparin intolerance [37,85,86].

#### 3.2.7. Post-Partum Use and Lactation

After discontinuation for delivery, danaparoid was either restarted or—in a few cases—initiated post-partum (233 of the pregnancy case reports). Fourteen women are known to have breast-fed their baby without any problem. Post-caesarean section use of danaparoid was investigated in 162 patients with no thromboembolic and 1 major bleeding event reported [87,88].

Breast milk samples from five women contained either no or small amounts of anti-Xa activity. Although this experience is limited, it appears that danaparoid is safe during lactation, especially because the small amounts of danaparoid secreted into the breast milk would be inactivated in the infant’s stomach [38].

#### 3.2.8. Children and Adolescents

Danaparoid use has been reported in 397 children (62 Caucasian and 335 Japanese) aged from a few weeks to 18 years. Thirty children received two distinct treatment episodes. Danaparoid treatment regimens (see Table 4) in 287 of these children, 51 of whom (all Caucasian) had HIT, are based on the multiple and many single patient reports [89,90,91,92,93] with clear dosing details. However, no specific paediatric dosing-finding studies have been reported.

The dosing intensity in non-Japanese children used was initially determined by the child’s body-weight (in kg), carefully balancing the thrombotic and bleeding risks; but, in the year 2001, a dosing schedule starting with an IV bolus of 30 U/kg followed by infusion of 1.2 to 2.0 U/kg/h (equivalent to 29–48 U/kg/day) in order to obtain the desired steady-state target anti-Xa levels of 0.4 to 0.8 U/mL was suggested. This schedule was later included in the 2008 guidelines of the Sixth American College of Chest Physicians Consensus Conference on antithrombotic therapy in neonates and children [94], as shown in Table 4.

Plasma anti-Xa activity monitoring should be performed at steady-state, i.e., at any time during the maintenance therapeutic infusion, or 3–4 h after an s.c. injection, or 10–15 min after an i.v. injection. It was reported for only 20 non-Japanese children (usually with HIT), and these levels were within the desired prophylactic or therapeutic target ranges (the same for children and adults). In some children, the total daily dose exceeded the recommended maximum dose because they had a higher-than-normal age-related body-weight.

#### 3.2.9. Danaparoid Dosing in Additional Paediatric Clinical Settings

Four subjects aged 6–15 developed HIT and valve thrombosis following open heart surgery [89]. All thromboses resolved with danaparoid infusions of 750 U twice daily for 10 days.

#### 3.2.10. DIC and Hepatic Thrombotic Syndromes

In the year 2000, danaparoid was approved for DIC treatment in Japan and later it was shown to have advantages over the serine protease inhibitors, e.g., gabexate mesylate and nafamostat mesylate [95]. It was also used off-label for hepatic thrombotic disorders [44], e.g., prevention (n = 69) or treatment (n = 538) of portal vein thrombosis (PVT) in adults [96,97,98,99,100], and for prevention of hepatic venous obstructive disease (VOD, also called Sinusoidal Obstruction Syndrome—SOS) and transplant associated thrombotic microangiopathy (TA-TMA) [90,91,92,93,101,102,103,104] after stem-cell transplantation in 197 adults and 326 children. VOD and TA-TMA optimally require both the antithrombotic and anti-inflammatory actions of danaparoid but, in most of the above reports, it was administered as two i.v. bolus injections or short infusions per day. These intermittent dosing schedules provide therapeutic daily doses of danaparoid but with peaks and troughs such that, prior to the next dose, the protective, immune modulatory component (the NA–HS) has been cleared from the circulation, leaving only a reduced antithrombotic effect of the HA–HS. Therefore, for these potentially fatal disorders, it is recommended that danaparoid should be administered as a continuous i.v. infusion that not only reaches earlier steady-state PK, but provides the combined anticoagulant effects of the NA–HS and the HA–HS with the anti-inflammatory actions of danaparoid NA–HS constantly throughout the infusion. In children up to 10 years, the loading i.v. bolus should be 750–1250 U and the two initial step-down infusions should be limited to 200 U/h for 4 h, and then 150 U/h for 4 h prior to the maintenance infusion. For all ages, the maintenance rate should be 5 U/kg/h to a maximum of 200 U/h with the same target range of plasma anti-Xa activity as for routine adult therapeutic infusions, i.e., 0.4–0.8 U/mL.

### 3.3. Danaparoid Transition to Long Term Anticoagulants

After successful therapeutic danaparoid treatment, i.e., thrombosis stabilisation, no increased bleeding risk and in HIT patients presenting with a sustained rise in platelet count, the patient may either be switched first to s.c. doses of danaparoid (using the schedule for routine DVT prophylaxis 750 units s.c. every 8–12 h (see Table 2)), and then to an oral anticoagulant, or switched directly to an oral anticoagulant.

#### 3.3.1. Switch to VKA

In patients with current HIT, switching to a vitamin K antagonist (VKA) may be associated with the risk of coumarin necrosis, induced by the rapid decrease of circulating protein C. Thus, it is critical for the patient to have reached a stable condition before initiating a VKA. Therefore, the platelet count should have persistently increased to the normal range (>150 × 10^9^/L) and any thrombotic process, present at danaparoid initiation or which had developed during treatment, is under control.

For direct transition from the i.v. infusion to VKA, the maximum danaparoid infusion rate should not exceed 200 U/h. As soon as the INR is within the therapeutic range (i.e., INR 2.0–3.0) for two consecutive measurements 24 h apart, the danaparoid infusion can be discontinued. If bleeding occurs during the transition period and the INR is not yet in the target range, then the danaparoid infusion should be stopped while continuing the VKA. If bleeding continues then omit the next VKA dose and restart 48 h later at the same dosing level as before.

#### 3.3.2. Switch to a DOAC

There are currently only sporadic reports of a switch to DOAC therapy directly after danaparoid treatment: Five acute HIT patients [105] switched to rivaroxaban 3 to 8 days after platelet count recovery on therapeutic-intensity danaparoid (four patients on maintenance infusions of 150–300 U/h and one patient on 2000 U s.c., b.d.). In four patients, the infusion was discontinued and rivaroxaban started when the plasma anti-Xa activity dropped to 0.5 U/mL; for the patient on s.c. injections, danaparoid was discontinued and rivaroxaban started at the time when the next injection would have been given. The initial treatment with rivaroxaban was either 15 mg b.i.d. (later 20 mg q.d.) in three patients or 20 mg q.d. constantly. No adverse events were reported.

Twenty patients with portal vein thrombosis (PVT) were switched from danaparoid treatment (1250 U i.v., b.d. for 2 weeks) to edoxaban [99] for long-term home treatment (60 or 30 mg o.d. depending upon their renal function). The patients all had oesophageal and/or rectal varices that did not bleed during their danaparoid treatment, but three patients on low-dose edoxaban developed gastrointestinal bleeding thereafter.

Five danaparoid-treated COVID-19 infected patients [45,106,107,108,109] were switched to apixaban (4), dabigatran (1), and to an unspecified DOAC (1).

With that limited evidence, it would seem prudent to follow the precautions used to switch from danaparoid to a VKA: one should wait until there is clinical stabilisation and persistent recovery of the platelet count. If the patient is receiving an i.v. maintenance infusion (Table 1, regimen 2a), with anti-factor Xa activity within the target range, and no bleeding risk, the infusion can be stopped in the morning or evening and the DOAC started 12 h later. If there is a bleeding risk, the danaparoid infusion should be switched to a subcutaneous regimen 1 (750 units s.c. every 8–12 h). When the plasma anti-Xa activity is <0.5 U/mL, danaparoid can be discontinued and the DOAC can be started instead of the next scheduled injection of danaparoid.

In patients considered to be at high thromboembolic risk and low bleeding risk, the initial higher dose of apixaban or rivaroxaban can be used. In patients with arterial thromboembolism, the maintenance dose of DOACs should be used.

## 4. Potential Adverse Events During Treatment

### 4.1. Delayed Platelet Count Recovery or New/Extension of Thrombosis

After pretreatment heparin discontinuation, the platelet count may continue to fall or remain low in many patients as a result of residual heparin in the circulation [17,18,19], especially if the danaparoid dosing intensity is inadequate. Furthermore, a covert pretreatment thrombosis may become clinically evident within the first 72 h of danaparoid treatment [59].

These complications during danaparoid treatment are commonly assumed to be due to cross-reactivity of danaparoid with the HIT-antibodies and hence considered a treatment failure. Danaparoid may therefore be discontinued, even in the absence of a (positive) cross-reactivity assay. However, because of the risk of changing to a less suitable alternative, other reasons for presumed treatment failure should be considered before stopping danaparoid. For example:

∘ The patient could have been underdosed because no overt clinical thrombosis was observed at HIT-presentation. Thus, danaparoid treatment was either initiated with a lower therapeutic i.v. infusion regimen without the (full) loading i.v. bolus or with the s.c. prophylactic regimen.
∘ The patient may have been re-exposed to a heparin.
∘ The patient may have an unrecognized co-morbid thrombophilic and/or thrombocytopenia-inducing disorder.
∘ The patient might be receiving a non-heparin co-medication that increases the risk of thrombosis and/or thrombocytopenia.

Thus, it is important to check for clinically asymptomatic thromboembolism by vascular ultrasound of the limbs, preferably before the switch to danaparoid, or as soon as possible thereafter; initiate treatment with the full i.v. bolus and therapeutic infusion regimen in all current HIT patients and look for the more common alternative possibilities to explain thrombosis an/or thrombocytopenia, e.g., heparin administration, sepsis or severe infection, anti-phospholipid syndrome, TTP/ITP, co-medication, etc. If danaparoid failure is still suspected, especially after the first 72 h of treatment, a functional test for danaparoid cross-reactivity should be performed.

If a new danaparoid cross-reactivity test is positive after 72 h of danaparoid treatment, the plasma anti-Xa activity is in the appropriate target range, and there is no evidence of heparin re-exposure or alternative disease or drug cause for the continued thrombocytopenia and/or thrombotic problem, then danaparoid cross-reactivity is possible and it should be discontinued. The patient should then be treated according to local standard practice with an alternative anticoagulant.

### 4.2. Occurrence of Bleeding

The low platelet count in HIT patients is not per se a risk factor for bleeding since these platelets are strongly activated. However, both HIT and non-HIT subjects may have a high bleeding risk due to a recent haemorrhage, an unstable post-operative/post-traumatic state, an intra-cranial lesion or vascular malformation, the presence of tracheotomy or pleural or bladder catheters, or hepatic dysfunction. Hepatic cirrhosis is not necessarily associated with an increased bleeding risk unless accompanied by portal hypertension with varices [110]. If such patients require therapeutic danaparoid treatment, the i.v. loading bolus injection should be halved and continued with the therapeutic infusion regimen whilst monitoring the patients carefully for bleeding. The i.v. loading dose should not be entirely omitted since this results in a dip in danaparoid effects (as shown by plasma anti-Xa measurements) until the steady state is reached more than 24 h later (see Supplementary Figure S1). During this dip, the patient is at an increased risk of thrombosis.

If bleeding occurs or recurs, then the instructions for danaparoid treatment modification given under ‘Non-Iatrogenic Bleeding’ below should be followed.

### 4.3. Bleeding During Danaparoid Treatment of Non-HIT or HIT Patients

#### 4.3.1. Iatrogenic Bleeding

Sometimes the cause of severe bleeding during surgery, invasive procedures, or medical treatment is unrelated to danaparoid, although it may prolong and/or worsen it.

Vascular injury during an invasive procedure or surgery, e.g., vessel laceration or inadequate vascular or bypass closure may cause bleeding. If this occurs, then danaparoid should be transiently discontinued until the vessel has been repaired and haemostasis is restored (if a maintenance infusion was being used prior to the operation/invasive procedure, then it can be restarted without a loading dose when it is considered safe to do so).

In case of bleeding associated with concomitant medications, for example with antibiotic treatment, danaparoid should be transiently stopped until the bleeding has ceased. If the patient has been on a maintenance infusion when the bleeding started, then this can be restarted without a loading dose, but at a 30% reduction in infusion rate. If the subject has sustained an accidental traumatic bleed (after a fall, etc.) then danaparoid should be transiently discontinued until the bleeding has stopped. If on a maintenance infusion, this should be restarted without a loading dose when it is considered safe to do so.

#### 4.3.2. Non-Iatrogenic Bleeding

If major bleeding occurs and iatrogenic causes have been excluded, the danaparoid infusion should be stopped for 6–12 h (at least until the bleeding stops), after which the danaparoid s.c. injections or infusion can be restarted without a loading dose. If bleeding recurs, then the procedure should be repeated without a loading dose and with a 30% reduction of the s.c. dose or infusion rate. If further bleeding occurs, then discontinue danaparoid and treat the subject according to local standard of practice.

If the plasma anti-Xa activity is ≥0.4 U/mL, the danaparoid infusion should again be stopped for 6–12 h (at least until the bleeding stops), after which subcutaneous danaparoid or danaparoid-infusion can be restarted without a loading dose and with a 30% reduction in infusion rate. If on an infusion and no further bleeding occurs but the plasma anti-Xa activity falls below 0.4 U/mL, then the infusion rate should be increased by 20% daily increments until the patient has reached the target range.

If a danaparoid treated patient develops only minor bleeding, then infusion should be reduced by 20%, or the next s.c. injection should be omitted.

#### 4.3.3. Danaparoid Overdose

Deliberate overdosing by self-injection of multiple Orgaran^®^ ampoules has been reported without incident. A one-year old child with Moyamoya disease was exposed to 350 U/kg/day (about 5 times the therapeutic maximum daily adult dose) on two separate occasions for 2 and 6 weeks [111]. No bleeding problem occurred despite the child undergoing intracranial surgery during treatment.

## 5. Discussion

### 5.1. Danaparoid in Perspective

Danaparoid is a complex natural product both with regard to its isolation and to its final chemical composition. Manufacturing the final product to approved specification limits ensures that the ratio of the HS to other components and, within the HS, the ratio of HA to NA subfractions remains relatively constant. Maintaining this consistency through frequent changes of license ownership has led to restricted availability in many countries and a high price. It has also greatly limited its clinical development. Additionally, danaparoid use is often erroneously avoided because potential investigators consider it has no clinically validated antidote, misunderstand the meaning of the plasma anti-Xa activity half-life, the level and significance of its cross-reactivity with anti-heparin antibodies, and its bleeding capacity. Some consider that it can only be administered intravenously. The mixture of mainly heparan sulphates that constitute danaparoid provide it with interactive, multifunctional anticoagulant and anti-inflammatory actions. This clinical advantage means that, apart from routine thrombosis prophylaxis, it is particularly important that treatment is conducted whenever possible, not only at a therapeutic dosing intensity, but it should be administered as a continuous i.v. infusion. The infusion (unlike intermittent injections) maintains the two most important components of danaparoid (with high (HA–HS) and no-affinity (NA–HS) for AT) in an optimal balance. This allows these two components to interact optimally to provide the most protective effect(s) for the patient. With an intermittent 12 and even 8 hourly injection schedule, this is impossible, since their greatly different rates of clearance from the circulation (24.5 h vs. 3.5 h respectively) leads to a rapidly decreasing HA–HS:NA–HS ratio. Thus, by the time of the next injection, whether an s.c. or i.v. schedule is being used, not only is there a trough in anticoagulant activity, but the protective effect of the NA–HS component is virtually absent. Underdosing is an important consideration particularly in patients with ongoing HIT because prophylactic danaparoid doses can increase PF4 binding to platelets; thus, further increasing the potential for danaparoid cross-reactivity. This is possibly why, in acute HIT patients on a prophylactic dosing regimen, the platelet count fails to recover, and re-thrombosis/thrombosis extension may occur. Hence, not only is the dosing intensity critical, but the administration route is also critical for successful treatment with danaparoid. It is also important to note that the units used for danaparoid dosing are not equivalent to the units used for the heparins, and routine clotting tests (PT, TT, APTT, and ACT) are hardly affected, even at recommended therapeutic dosing intensities. Thus, neither international heparin standards for the plasma anti-Xa activity assay nor routine clotting tests can be used either to indicate or adjust the required dosing intensity of danaparoid.

Extensive use of DVT as a prophylaxis in elderly patients (<65 years), as well as its ability to restore and maintain patency in extracorporeal circuits required to manage patients in renal failure, its use in pregnancy, in children to prevent hepatic thrombotic complications during treatment for haematogenous cancers, and in (auto)-immune disorders have shown it to be safe and effective. An additional advantage, because it depends for its antithrombotic action on two different modes of attack on the clotting cascade, is that danaparoid does not appear to affect plasma AT levels and appears to be less reliant upon plasma AT activity (except at very low levels) than the heparins.

The current article summarises the available published dosing experience with danaparoid and provides additional advice for the practicing physician treating these complex patients. These dosing recommendations and advice for management of approved and off-label indications and other clinical challenges are sufficiently flexible to allow the physician to adapt them according to the risk/benefit profile of the patient under treatment.

Danaparoid is not a simple product either with regard to its isolation or to its final chemical composition. Along with the frequent changes of license ownership, this has led to restricted availability in many countries, a high price, and has also greatly limited its clinical development. Additionally, danaparoid use is often erroneously avoided because potential users are concerned about the following:

- the lack of a clinically validated antidote;
- its long plasma anti-Xa activity half-life for safety reasons despite it being only a surrogate for danaparoid as a whole;
- its potential for cross-reactivity with anti-heparin antibodies;
- bleeding;
- its use in patients with renal or hepatic dysfunction.

Yet, the same arguments apply to fondaparinux and some of the newer DOACs that currently dominate the antithrombotic market. Some of these fears arise because publications from Asia are rarely accessed unless they are in English. Thus, most of the Japanese literature concerning danaparoid studies remains uncited in treatment guidelines and reviews devoted to the same clinical indications [112,113,114,115]. However, with a TGI activity half-life of about 7 h, rare clinical consequences of its low potential for cross-reactivity with HIT antibodies, and a low bleeding-inducing capacity (with a possible antidote in rFVIIa [34,54]), danaparoid has been shown in a large number of patients over four decades to provide effective, well tolerated, and safe anticoagulation for both its approved indications of HIT, DVT prophylaxis and treatment of DIC, and for the prevention or treatment of serious off-label indications, including patients in renal and hepatic failure. It has been successfully used in the most challenging intensive care patients, often requiring an antithrombotic and/or anti-inflammatory action. This broad experience provides assurance for treating physicians that danaparoid is both safe and efficacious. The available evidence from this broad experience provides assurance to the treating physician. Thus, danaparoid when dosed appropriately, can continue to find a widening role in the management of many serious disorders. However, the lack of adequately powered, blinded CRTs between danaparoid and any of the newer antithrombins and DOACs is to be regretted, and these limitations have combined to prevent full investigation and appreciation of its combined antithrombotic and anti-inflammatory/immune modulatory effects.

### 5.2. International Guideline Recommendations

Several international Guidelines review the therapeutic options in HIT and provide recommendations on the use of danaparoid. For patients with normal renal function and HIT with thrombosis, the American College of Chest Physicians (ACCP) guidelines [6] suggest argatroban, lepirudin, or danaparoid over other non-heparin anticoagulants (Grade 2C). For patients with isolated HIT (HIT without thrombosis), the use of lepirudin, argatroban, or danaparoid is recommended over the further use of heparin, LMWH, or initiation/continuation of a VKA (Grade 1C). For patients with normal renal function, the use of argatroban, lepirudin, or danaparoid is suggested over other non-heparin anticoagulants (Grade 2C). For patients with acute or subacute HIT who require renal replacement therapy, the use of argatroban or danaparoid is suggested over other non-heparin anticoagulants (Grade 2C). For pregnant patients with acute or subacute HIT, use of danaparoid is suggested over other non-heparin anticoagulants (Grade 2C). Only if danaparoid is not available is the use of lepirudin or fondaparinux suggested (Grade 2C).

For patients with acute HIT complicated by thrombosis or acute HIT without thrombosis (isolated HIT), the American Society of Hematology (ASH) guideline panel [8] recommends discontinuation of heparin and initiation of a non-heparin anticoagulant (strong recommendation, moderate certainty in the evidence about effects). When a non-heparin anticoagulant is being selected, the ASH guideline panel suggests argatroban, bivalirudin, danaparoid, fondaparinux, or a direct oral anticoagulant (DOAC) (conditional recommendation, very low certainty in the evidence). For patients with acute HIT, who are receiving renal replacement therapy and require anticoagulation to prevent thrombosis of the dialysis circuitry, treatment with argatroban, danaparoid, or bivalirudin, rather than other non-heparin anticoagulants, is suggested (conditional recommendation; very low certainty in the evidence about effects). For patients with remote HIT, who require VTE treatment or prophylaxis, administration of a non-heparin anticoagulant (e.g., apixaban, dabigatran, danaparoid, edoxaban, fondaparinux, rivaroxaban, or VKA) rather than UFH or LMWH is recommended (strong recommendation, very low certainty in the evidence about effects [8].

The guidelines from the European Society for Vascular and Endovascular Surgery [116] recommend the use of alternative anticoagulants in suspected or confirmed HIT. Non-heparin anticoagulants that have been used in HIT include argatroban, bivalirudin, desirudin, danaparoid, and fondaparinux. However, the authors emphasize that only argatroban and danaparoid are currently available and licensed for acute HIT [116].

## 6. Conclusions

After more than 40 years of danaparoid use in patients with HIT and associated co-morbid conditions, or for patients who cannot be treated with heparin for various reasons, a wealth of evidence has been accumulated both from clinical trials, a plethora of single and multiple case reports, and from the compassionate use program of danaparoid. Therefore, dosing recommendations can now be given for a broad spectrum of clinical indications, based on an interdisciplinary consensus. Nevertheless, careful judgement is required for each individual patient, especially in high-risk situations, including HIT-Syndrome. Further analysis of available evidence from various sources will further strengthen and broaden clinical applications of danaparoid.

## Figures and Tables

**Table 1:** Infusion rate for iv-Infusion of danaparoid from a stock solution of 9 U/mL.

Danaparoid Dosing Required	Infusion Rate of the Stock Solution	Danaparoid Dosing Delivered
400 U/h	44 mL/h	396 U/h
300 U/h	33 mL/h	297 U/h
200 U/h	22 mL/h	198 U/h

**Table 2:** A summary of danaparoid treatment regimens for approved Indications.

Clinical Purpose	Treatment Schedule	Regimen No. ^2^
Medical Prophylaxis for Non-HIT & Remote HIT patients ^1^	≤90 kg: 750 U b.i.d, s.c. >90 kg: 750 U t.i.d. or 1250 U b.i.d., s.c. Between days 7 and 14 the transition to a VKA can be made	**1a**
Pre-operative dose Peri-operative thromboembolic prophylaxis in Non-vascular surgery for Non-HIT and HIT patients Post-operative dosing	Patient is already on therapeutic i.v. danaparoid infusion and needs emergency surgery Stop the infusion 12 h prior to surgery or Patient is not already on danaparoid treatment or is on prophylactic danaparoid prior to surgery, i.e., ≤90 kg: 750 U b.i.d, s.c. or >90 kg: 750 U t.i.d. or 1250 U b.i.d., s.c. Then give the last or only pre-op dose ≥ 6 h prior to surgery. The first post-operative dose is given, when haemostasis is established but not earlier than 6 h post-operatively. ≤90 kg: 750 U b.i.d, s.c. for up to 14 days >90 kg: 750 U t.i.d. or 1250 U b.i.d., s.c. for up to 14 days If danaparoid is (still) required at therapeutic dosing levels post-operatively, then restart the infusion at the pre-operative maintenance infusion rate without a bolus injection when haemostasis is achieved, but no sooner than 6 h post-operatively (see below [Regimen 2a] for maintenance infusion adjustments).	**1b**
Therapeutic dosing for current HIT and for continuous renal replacement therapy (CRRT) in the absence of clotting in Non-HIT or HIT patients	i.v. loading bolus as follows injected over 5–10 s: 2250 U i.v. for patients 55–90 kg body weight, 1500 U i.v. for patients < 55 kg body weight, 3750 U i.v. for patients > 90 kg body weight, Plus, in all patients an immediate ‘step-down’ i.v. infusion of 400 U/h for 4 h, then 300 U/h for 4 h then 150–200 U/h maintenance infusion for 7 days or longer if required. In patients with impaired renal function (i.e., eGFR < 30 mL/min/1.73 m^2^ and/or patients on CRRT) the maintenance dose should be reduced to 150 U/h. The maintenance infusion rate can be adjusted according to the risk of bleeding. If monitored at steady-state within 6 h after start of treatment the plasma anti-Xa activity level should be between 0.4 and 0.8 anti-Xa U/mL. If the plasma anti-Xa level is outside this range or, in the absence of monitoring, thrombosis or bleeding occur then the maintenance infusion rate can be adapted accordingly by increase or decrease of 20% of the maintenance infusion rate.	**2a**
CRRT use in case of clotting of the circuit in Non-HIT and HIT patients	2250 U i.v. bolus, +step-down i.v. infusion of 400–600 U/h for 4 h, then 300 U/h for 4 h, then 100–400 U/h (maintenance rate) ^3^ The aim is to reach the lowest rate that achieves and maintains circuit patency. High infusion rates within the range may be initially required if circuit clotting has been a problem or the patient recently received a high heparin dose. Once under control, the rate can be adjusted as indicated above for Regimen 2a depending upon whether it is only needed to maintain circuit patency or to provide systemic TE prophylaxis or treatment in a patient at high risk or affected.	**2b**
Vascular surgery/invasive vascular procedure in Non-HIT and HIT patients ^4^ Pre-operative/procedure Intra-operative/procedure Post-operative/procedure	≤90 kg: 2250 U i.v. bolus pre-procedure/surgery >90 kg: 3750 U i.v. bolus Depending upon the length and complexity of the surgery additional 1500 U boluses or a continuous infusion of 150–200 U/h may be used. If needed, then no less than 6 h post-operatively (with adequate haemostasis) restart the infusion at 150 U/h for 5–7 days. The maintenance infusion rate can be adjusted according to the risk of bleeding or thrombosis. If monitored at steady-state within 6 h of starting it the plasma anti-Xa activity level should be 0.4–0.8 U/mL. If outside this range or if, in the absence of monitoring, thrombosis or bleeding occur then the maintenance infusion rate can be increased or decreased by increments of 20%.	**3** **(2a)**
Intermittent haemodialysis (HD) in Non-HIT and HIT patients HD not daily HD daily	If plasma anti-Xa monitoring is available Bolus pre-dialysis for the first 2 dialyses: 2250 U i.v. if patient is <55 kg 3750 U i.v. if patient is ≥55 For the 3rd and subsequent dialyses, the pre-dialysis dose is dependent upon the pre-dialysis anti-Xa activity of the previous dialysis: <0.3 U/mL 3000 U i.v. pre-dialysis (<55 kg: 2000 U) 0.3–0.35 U/mL: 2250 U (<55 kg: 1500 U) 0.35–0.4 U/mL: 2000 U (<55 kg: 1500 U) >0.4 U/mL: danaparoid not required for that dialysis, but the pre-dialysis plasma anti-Xa activity level will determine the dose required for the following dialysis As above, but 2250 U (<55 kg: 2000 U) prior to 2nd dialysis. By the 4th–6th dialysis the dose will have reached a steady state and will be the same for subsequent dialyses, however it is advised to monitor for bleeding or circuit clotting/systemic thrombosis If there is a significant risk of systemic thrombosis or a thrombosis is evident, then use the continuous infusion of Regimen 2a instead. However, since renal failure may lead to accumulation, the maintenance infusion rate should be 150 U/h, as described above, to avoid plasma anti-Xa activities > 0.8 U/mL.	**4** **(2a)**
Flush doses for all patients	750 U danaparoid diluted into 50 mL saline. 5–10 mL of this solution to flush intravascular lines/access ports as required	**6**

^1^ HIT-Ab not detectable by EIA or functional test; ^2^ the splitting of regimens 1 and 2 into two sub-indications is for simplification in this document only; ^3^ this schedule is used for each CRRT session. If routine filter changing every 24 h is performed the maintenance infusion rate should be continued, if there has been no clotting in the circuit, (i.e., no new loading i.v. bolus is required); ^4^ PCI, cardiac catheterisation, angiography, IVCF, or IABP insertion/removal.

**Table 3:** Danaparoid Dosing and Duration in Pregnancy and Post-Partum (PP).

Danaparoid Treatment	Pregnancy and/or Post-Partum
Total daily dose during pregnancy i.v. and/or s.c: 1000–2250 U >2250–3000 U >3000 U	Total 137 ^1^ 78 58.6% 46 34.6% 24 18.0%
Duration of danaparoid treatment (weeks) in pregnancy: Median Range	18 (92) ^2^ <1–39
Duration (days) after re-starting danaparoid post-partum ^3^: Median Range	28 (33) ^2^ 2–63
Duration (days) danaparoid used post-partum only ^4^: Median Range	2 (144) ^2,^ 1–4

^1^ Dosing for 137 known, some received >1 dose regimen; ^2^ bracketed numbers are patients with information; ^3^ following use during pregnancy; ^4^ i.e., no antenatal use of danaparoid.

**Table 4:** Published Dosing for children aged < 1–18 years.

Treatment Indication	Age (Years)	Daily Dosing ^1^	Target Plasma Anti-Xa Activity *
Arterial and Venous Thromboprophylaxis	≤2	10–15 U/kg b.d., s.c. to a maximum of 1250 U/day	0.1–0.4 U/mL
	9–17	10–30 U/kg b.d., s.c. to a maximum of 2250 U/day	0.1–0.4 U/mL
Hepatic thrombotic syndromes ^2^	1–18	15–30 U/kg b.d., s.c. or i.v. to a maximum of 2250 U/day	0.1–0.4 U/mL ^2^
General Thrombosis Treatment	≤2	30 U/kg i.v. loading bolus then 6 U/kg/h i.v. infusion for 4 h to maximum 300 U/h then 4 U/kg/h i.v. infusion for 4 h to maximum 200 U/h then a maintenance infusion 2.5 U/kg/h to maximum 1500 U/day	0.4–0.8 U/mL
	9–17	30 U/kg i.v. loading bolus to maximum 2250 U then 6 U/kg/h i.v. infusion for 4 h to maximum 400 U/h then 4 U/kg/h i.v. infusion for 4 h to maximum 300 U/h then a maintenance infusion 3 U/kg/h to maximum of 3000 U/day (but if >55 kg then up to maximum 4800 U/day)	
Pre-Cardiac catheterization	≤2	60–120 U/kg i.v.b. to maximum 1500 U	0.4–0.8 U/mL post bolus
Intermittent (not daily) haemodialysis ^3^	2–10	1st and 2nd dialysis: a pre-dialysis i.v. bolus 30 U/kg + 1000 U to a maximum of 2250 U/dialysis.	0.4–0.8 U/mL during dialysis
	10–17	1st and 2nd dialysis: a pre-dialysis i.v. bolus 30 U/kg + 1500 U to maximum of 3000 U/dialysis	
Continuous ambulatory peritoneal dialysis	≤2–17	5–15 U/kg either s.c.in divided doses or as an i.v. infusion (0.2–0.6 U/kg/h) to maximum 1000 U/day	Should not exceed 0.4 U/mL

* At steady state, or within 15 min of an i.v. bolus or 3–4 h after an s.c. bolus.; ^1^ the maximum daily dosing for each clinical indication is a guiding value. It is expected that the dose required to maintain circuit/filter patency is less than this. ^2^ This includes Venous Occlusive Disease (also known as Sinusoidal Obstructive Syndrome or SOS) and Transplant Associated Thrombotic Microangiopathy (TA-TMA); ^3^ for the third and subsequent haemodialysis see Table 2.

## Supplementary Materials

The following supporting information can be downloaded at: https://www.mdpi.com/article/10.3390/ph17121584/s1, Table S1. Danaparoid dosing in relation to body weight stratification. Figure S1. Both the loading bolus and the higher step-down initial short infusions ensure that steady-state danaparoid levels are achieved immediately (red line). Without the step down infusions even a higher i.v. loading bolus does not prevent the dip in danaparoid activity within the first 24 h of the infusion maintenance infusion (blue line).
