# Supplementary material for: Danaparoid—Consensus Recommendations on Its Clinical Use
Source: Pharmaceuticals (Basel). 2024 Nov 25;17(12):1584. doi: 10.3390/ph17121584 (PMC11677338; doi:10.3390/ph17121584)
Supplement: Supplementary file 1 [file pharmaceuticals-17-01584-s001.zip › pharmaceuticals-3033411-supplementary.pdf]

pharmaceuticals

Supplementary Materials:

Table S1. Danaparoid dosing in relation to body weight stratification.

Body weight (Kg)	Patients N	I.V. bolus (U)		Maintenance Infusion rate/h (U)		s.c. dose/day (U)	
		mean	range	mean	range	mean	range
<55	47	1470	750 - 2500	152	15 - 375	2227	1000 - 3000
55-90	303	2258	500 – 3750	190	75 - 400	2097	1000 - 7500
>90	89	2669	1250 – 3750	210	100 - 400	2354	1500 - 5250

Correlation coefficients for body weight range vs ivb = 0.9357, vs maintenance infusion rate = 0.9368 and s.c. dosing =0.2358.

The Therapeutic Danaparoid Infusion (Regimen 2a)

Plasma anti-Xa activity response during the initiation and subsequent maintenance danaparoid infusion are shown in Suppl. Figure 1.

Continuous infusion of danaparoid has three principle advantages:

- It allows all components of danaparoid to be present in the circulation at all times. This is important since the elimination half-lives of the components varies from 2 – 25 hours but their effects - anticoagulant and anti-inflammatory/immune modulation, depend on interaction between them. This interaction is lost or greatly diminished if danaparoid is administered as intermittent b.d. or s.c. bolus injections since the short-life components are rapidly removed from the circulation.
- It allows faster attainment of the target range.
- TE treatment and anti-inflammatory/immune modulation actions have been shown to be most effective at therapeutic dosing intensities which are more comfortably achieved (for the patient) by an i.v. infusion.

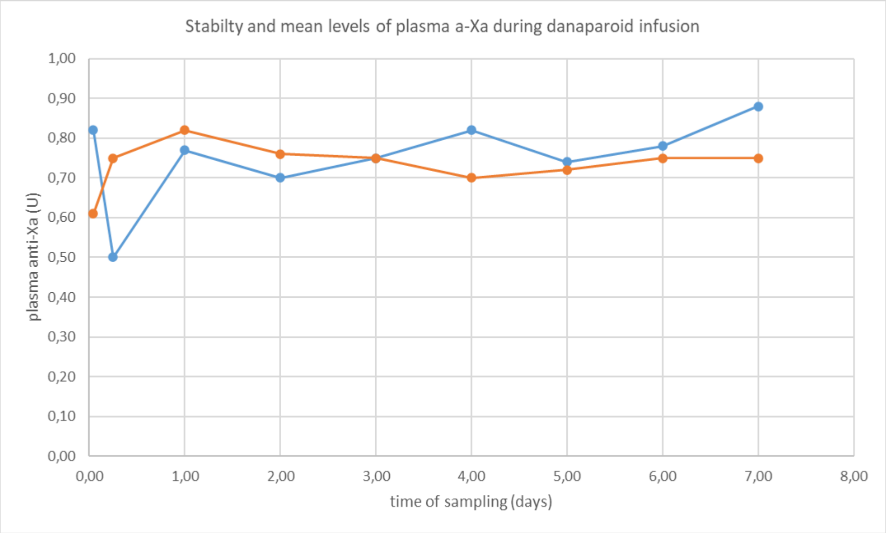

Figure S1. Figure Legend: Both the loading bolus and the higher step-down initial short infusions ensure that steady-state danaparoid levels are achieved immediately (red line). Without the step down infusions even a higher i.v. loading bolus does not prevent the dip in danaparoid activity within the first 24 h of the infusion maintenance infusion (blue line).

The Danaparoid Loading Dose

The loading bolus is essential to Regimen 2a since without attainment of steady-state danaparoid PK is delayed producing a dip in effect[43] (blue line in Fig. 1) that increases the risk of early thrombotic events and in HIT patients failure to neutralise the effect of the HIT-Ab within the first 24 h after treatment initiation. The loading dose and the subsequent 'step-down' danaparoid infusions prior to the maintenance overcome this problem (red line Fig. 1). Hence unless there is a convincing reason, it should not be omitted but it can if necessary be reduced, e.g. if the bleeding risk is high and/or the patient recently had a spinal/epidural catheter removed.
